# In Search of Autism’s Roots

**DOI:** 10.1371/journal.pbio.2000958

**Published:** 2016-09-30

**Authors:** Liza Gross

**Affiliations:** Public Library of Science, San Francisco, California, United States of America

Until Dustin Hoffman’s uncanny performance as an autistic savant in *Rain Man*, few outside medical circles knew much about autism. Hoffman’s humane portrayal of a socially inept man prone to nervous tics and obsessive ruminations, punctuated by stunning feats of math and memory, challenged us to accommodate people with special needs and reconsider our notions of normalcy. (For the record, only about one in ten people with autism are savants.)

**Fig 1:**
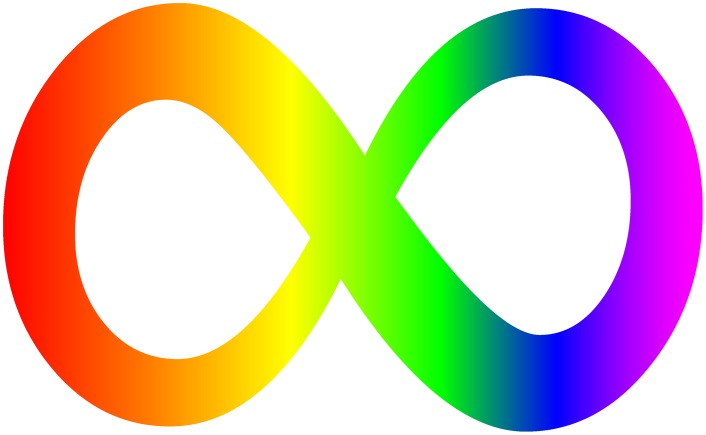
Many people with autism and their families like the rainbow infinity sign as a symbol of neurodiversity, representing not just the tremendous diversity of people on the spectrum but also of human cognitive and intellectual capabilities.

Such compassionate views of autistic people were hard to find a decade later, after the British gastroenterologist Andrew Wakefield unleashed a panic with a now thoroughly discredited, retracted 1998 paper that linked the measles virus in the measles, mumps, rubella vaccine to autism. Suddenly, autism became a parent’s worst fear [1]. Wakefield paved the way for a rotating roster of unsupported theories that linked vaccines to autism, and many parents stopped vaccinating their kids. Celebrities like Jenny McCarthy encouraged parents to see autism as far scarier than a deadly disease like measles and blamed an expanded vaccine schedule for skyrocketing autism rates. It didn’t matter that experts attributed the increase to clinicians’ heightened awareness and new diagnostic criteria that saw autism as a constellation of neurodevelopmental conditions, from the severely disabled to the highly gifted, now called autism spectrum disorder (ASD) (Fig 1).

Pediatricians hoped parents’ fear of vaccines—and corresponding stigmatization of autism—would finally subside when scientists figured out ASD’s causes. Unfortunately, unraveling the causes of such a complex range of conditions has proved challenging. Still, researchers have associated mutations in over 100 genes with ASD and have linked alterations in the structure and function of brain regions with autistic traits [2]. Many studies have focused on genes involved in the formation of synapses, the junctions between brain cells [3].

In a new study in *PLOS Biology* [4], researchers took an evolutionary approach to identify gene expression patterns during brain development that could contribute to autism. The team, led by Svante Pääbo, Schahram Akbarian, and Philipp Khaitovich, reasoned that since the cognitive abilities impaired in autism include those that appear to be uniquely human, it’s likely that autism arises from changes in patterns of gene expression that may be specific to humans.

To test this possibility, they measured gene expression patterns in the prefrontal cortex in humans, chimpanzees, and rhesus monkeys. The prefrontal cortex plays an important role in advanced cognitive functions that are affected in ASD, including language and social communication. The brain samples allowed the team to measure gene expression over most of the lifespan of each species, and then to identify differences in expression between autistic patients and controls. (All samples came from postmortem tissue.) In keeping with previous studies, the group saw that expression of synapse-related genes during cortical development peaked five to ten years later in humans than in chimpanzees and monkeys. But synaptic gene expression peaked much earlier in the autistic group than in the controls. And this modified expression pattern included many genes that have been implicated in autism by genetic studies.

These altered gene expression patterns disrupt brain development programs critical to our capacity for social learning and language, the authors argue. It’s not clear exactly how these disturbances might contribute to ASD, though many studies have linked impaired synapse formation and function to autism [5].

As clinicians have come to appreciate that an ASD diagnosis covers a wide range of needs and talents, they’ve also recognized that it can carry increased risk for several health problems, including epilepsy and bowel disorders. In a paper published in *PLOS ONE* in July [6], researchers at Stanford analyzed genes associated with ASD and these associated disorders to look for signs that the genes may have evolved at different rates. Interestingly, the authors report, genes that were uniquely involved in autism appear to evolve faster than other genes expressed in the brain, while those also involved in the coexisting conditions seem to evolve more slowly.

Autistic people also face a higher risk of anxiety disorders, including obsessive compulsive disorder [7], psychiatric disorders that can be obscured by communication problems [8], metabolic disorders, sleep disorders, immune disorders, and other ailments [9]. To help patients with diverse medical needs, researchers at hospitals and medical centers in four states developed an algorithm to scan electronic health records to identify coexisting conditions for over 20,000 people with autism. The study, published in July in *PLOS ONE* [10], offers a tool to help clinicians tailor treatment plans to the individual needs of ASD patients.

It’s hard to know for sure, but it appears that recent advances in understanding the biological underpinnings of autism may be convincing parents that vaccines have nothing to do with it. For years, polls showed that one in four respondents believed vaccines cause autism. Last year, Gallup reported that just six percent believe the myth. Hopefully, they’re also coming to see autism as a disability that can be accommodated, not a condition to be stigmatized and feared.

For more detailed reading, please see the associated PLOS Collection [11].
